# Semiconducting Metal Oxide Based Sensors for Selective Gas Pollutant Detection

**DOI:** 10.3390/s91008158

**Published:** 2009-10-16

**Authors:** Sofian M. Kanan, Oussama M. El-Kadri, Imad A. Abu-Yousef, Marsha C. Kanan

**Affiliations:** American University of Sharjah, Biology & Chemistry Department, P.O. Box 26666, Sharjah, UAE; E-Mails: oelkadri@aus.edu (O.M.E.-K.); iabuyousef@aus.edu (I.A.A.-Y.); mkanan@aus.edu (M.C.K.)

**Keywords:** sensors, metal oxides, pollutants, selective detection, gases

## Abstract

A review of some papers published in the last fifty years that focus on the semiconducting metal oxide (SMO) based sensors for the selective and sensitive detection of various environmental pollutants is presented.

## 1. Background

Semiconducting metal oxide sensors are one of the most widely studied groups of chemiresistive gas sensors. These sensors are designed to react with one class of gases whereby the SMO undergoes reduction and oxidation. This process causes the SMO sensors to exchange electrons with the target gas at a certain characteristic rate, thereby affecting the sensor's resistance and yielding a certain signal. The reaction of SMO materials with gases and the result of the conductometric changes were introduced in the early 1950's by Brattein *et al.* [1] and Heiland [2]. The direct applications of the SMO sensors as catalysts and electric conductive detectors toward various gases were then introduced by Bielanski *et al.* [3] and Seiyama *et al.* [4].

During the past few decades, SMO gas sensors have become a prime technology in several domestic, commercial, and industrial gas sensing systems. Three different types of solid state gas sensors are widely available nowadays [5,6]. These sensors are based on electrochemical behavior, catalytic combustion, or resistance modulation of SMO [6-14]. Among the available gas sensing methods, the SMO gas sensor devices have several unique advantages such as low cost, small size, measurement simplicity, durability, ease of fabrication, and low detection limits (< ppm levels). In addition, most SMO based sensors tend to be long-lived and somewhat resistant to poisoning. For these reasons, they have rapidly grown in popularity, becoming the most widely used gas sensors available these days.

Several materials are fabricated to enhance the sensing characteristics of the SMO gas sensors. Various SMO mixed with different dopants, catalysts, adhesives, binders, volatile fillers, and electrodes all have been studied [15-46]. In addition to the variations in the composition of the SMO materials, their film deposition methods provide another variable for sensor design. These deposition methods include pyrolysis, oxidation of metallic films, reactive sputtering, chemical vapor deposition (CVD), laser ablation, and electron-beam evaporation techniques [47-60]. This review article will focus on the principle and use of SMO sensors for several applications, for gas detection, and environmental monitoring. The article will also discuss several environmental influence factors that might affect a SMO sensor's performance in terms of sensitivity, selectivity, and response time.

## 2. Working Principle of SMO Gas Sensors

Despite the simplicity of SMO measurements for use as gas sensors, the detection mechanism is complex and not yet fully understood. This complexity is due to the various parameters that affect the function of the solid state gas sensors. These include the adsorption ability, electrophysical and chemical properties, catalytic activity, thermodynamic stability, as well as the adsorption/desorption properties of the surface [5,61-69]. However, it is believed that gas sensing by SMO devices involve two major key functions as receptor and transducer functions [70,71]. The former involves the recognition of a target gas through a gas-solid interface which induces an electronic change of the oxide surface, while the latter is based on the transduction of the surface phenomenon into an electrical resistance change of the sensor [70]. When a sensor is heated to a high temperature in the absence of oxygen, free electrons easily flow through the grain boundaries of the SMO film. In an oxygen atmosphere, oxygen is adsorbed onto the SMO surface, forming a potential barrier at the grain boundaries. The interaction of atmospheric oxygen with the SMO surface forms charged oxygen species, which trap electrons from the bulk of the material. The layer of charged oxygen at the surface repels other electrons from interacting with the bulk of the film, creating a region depleted of electrons which results in an increased potential barrier at the grain boundaries. This impedes the flow of electrons and thus increases the resistance. When the sensor is exposed to an atmosphere containing a reducing gas, the SMO surface adsorbs the gas molecules and lowers the potential barrier, allowing the electrons to flow easily and thus reducing the electrical resistance. In this manner, the sensors act as variable resistors whose value is a function of gas concentration.

Metal oxides exhibit various electro-physical features, ranging from insulators to wide band-gap semiconductors [72-84]. The non-transition metal oxides contain elements with one oxidation state because they require a large amount of energy to make other oxidation states that would bind to the oxygen ion ligand [72]. In contrast, because of the various oxidation states that might form on transition metal oxides compared to non-transition metal oxides, the surface properties and the types of chemisorptions that occur on the surface are important and have been widely studied [72,73,75]. This variation in the oxidation states causes significant changes in the surface chemistry response toward oxygen and other target gaseous molecules [5]. Despite the fact that transition metals of d^n^ oxides with n > 0 exhibit high potentials to perform oxidation and reduction processes, it has been noted that only transition metals with d^0^ configuration displayed real gas sensor application. For example, TiO_2_, V_2_O_5_, WO_3_ have d^0^ configurations and are the most widely used transition elements in sensor technology, along with non-transition elements with a d^10^ configuration like ZnO and SnO_2_ based materials. The above choice of metal oxides were found to have a filled valence band of predominantly oxygen 2p character with band gap ranges between 3–4 eV [77-84].

Since the mode of adsorption and/or reaction occur on a sensor's surface, several researchers have reported that the conductivity response is highly affected by the presence of an efficient catalyst that enhances the surface reactivity toward the target gaseous molecules [61,62,68,75,85-87]. In specifics, catalytic reactions involving surface oxygen can change both the surface potential along with its defect level and thus control the electro-physical properties of the nanocrystalline modified metal oxide. Therefore, tuning the surface characteristics with specific catalysts has resulted in major advances in sensor technology where both reactivity and selectivity in a material's responses were improved [88]. Both “spill over” and Fermi energy control mechanisms were applied to explain how catalysts affect the sensing strategy. In the “spill over” mechanism, the catalysts will dissociate the molecule and then the atoms will spill over the surface while in the Fermi energy mechanism the adsorbed oxygen will remove electrons from the catalyst and then the catalyst will effectively dislodge from the surface catalyst film.

## 3. Testing Setup, Film Deposition and Delivery System

**Figure 1.**
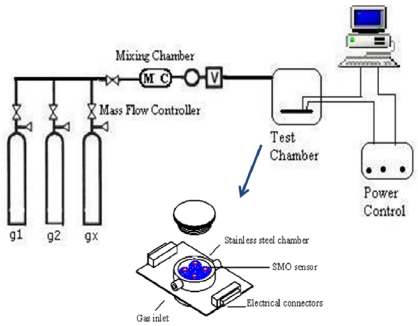
A general schematic for SMO gas sensor devices.

Despite the fact that the testing setups of SMO sensors tend to differ, their overall principle remains the same. Figure 1 shows a general schematic of a SMO gas sensor device.

As illustrated in Figure 1, the sensor array mainly consists of a target gas, a multi-component gas mixer, a mass flow controller unit, a testing chamber, a power supplier and heaters, and an electrometer for resistance measurement. LabVIEW based software is mainly used to control all testing parameters and measurements during the experiment. The testing chamber consists of SMO sensor platforms with the ability to control and measure each sensor's temperature and resistance. The SMO films are deposited on the sensing element as thin or thick film substrates. Thin film deposits are made via ultra high vacuum (UHV) or electron beam evaporation techniques, while thick films are deposited using spin coating methods or via direct deposition of the corresponding SMO suspension. The sensor platform is bonded into a standard header and then placed in a test chamber and annealed at 400 °C using a temperature controller prior to gas exposure where the testing experiments of the SMO to the target gaseous molecules begin.

## 4. Applications in Environmental Monitoring and Gas Detection

### 4.1. Nitrogen oxide gases (NOx)

Different carbon nanotube (CNT) films produced by a chemical vapor deposition (CVD) technique were tested as resistive NO_2_ sensors for environmental applications [89]. It was found that the CNT networks provide good response to low NO_2_ concentrations and excellent selectivity in the presence of interfering gases like NH_3_, H_2_, octane, and toluene. The pretreatment period, sensor response, and recovery times were all found to be temperature dependent. Moreover, the results suggest that CNT network sensitivity upon exposure to different gases can be conveniently tuned by suitably choosing the airbrushed CNT materials, and by simultaneously controlling both the CNT deposition rate and CNT transport properties. As a result, CNT films offer fascinating opportunities for their use as sensor materials [89].

The sensitivity of the CNT sensors was found to depend on the deposition methods. For example, using a pulsed laser ablation (PLA) method, where the graphite contains Ni and Co catalysts, the resistance of the CNT (single and multi walled CNTs) gas sensor decreased with an increase of ambient NO gas or NO_2_ gas concentration. It was also found that the temporal rate of change in the resistance was proportional to the concentration of the target gas and it can be useful for rapid estimation of the target gas concentration [90]. CNT films modified with SMO materials have been recently used to detect low concentrations of NOx gases at low temperature. For example, CNTs deposited with platinum or palladium nanoclusters (deposited via radio frequency plasma enhanced CVD) serve as very promising chemical nanosensors with high sensitivity, reversibility, and a very low limit of ppb detection of NO_2_ [91]. Moreover, it has been reported that CNTs mixed with hexagonal-WO_3_ composites were able to detect as low as 100 ppb of NO_2_, without having to heat the sensor substrates during operation. The detected concentration level is very close to the ambient air quality standard for nitrogen dioxide, which demonstrates the environmental applicability of the new gas sensors [92].

Tungsten oxide based materials have received a great deal of attention in the fabrication of SMO gas sensor devices. For example, several SMOs based on WO_3_ sensors [93-98] and WO_3_ modified with various metal composites [99-108] have been used for potential NO_x_ sensors. The reactivity of WO_3_ based sensors was found to be highly dependent on the deposition process and testing protocol [93-98]. For instance, films of nanostructured WO_3_ with high surface roughness were obtained using a modified thermal evaporation technique [93]. It was found that the sensors exhibit high responses, selectivity and short response times that are enhanced by decreasing the working temperature down to a minimum of 100 °C. At this temperature, high sensitivity was reached for NO_2_ with a detection limit lower than 100 ppb that caused a high variation in the film electrical resistance. Furthermore, the low responses obtained towards high concentrations of NH_3_ (10 ppm) and CO (400 ppm) suggest promising selective properties [93].

Recently, Yang *et al.* reported various synthetic methods for preparing efficient WO_3_ sensing elements for high temperature potentiometric NOx sensors [98]. Methods include deposition on Yttria-stabilized zirconia (YSZ) attached to two Pt and Pd wires (Sensor A), WO_3_ mixed with a-terpineol (Sensor B), a hydrogen peroxide/WO_3_ solution (sensor C), and WO_3_ deposition on YSZ followed by UV radiation and ozone treatment (Sensor D). The experimental results showed that the Pt electrode (Sensor A) had the lowest NO*_x_* signal compared to the other devices containing WO_3_ whereas, the WO_3_/YSZ sensing electrode fabricated by the UV-ozone treatment method (sensor D) had better mechanical stability, higher sensitivity, and better response/recovery times than devices fabricated from commercial WO_3_ powder [98]. Moreover, several studies have emphasized that grain size reduction in metal oxide films is one of the key factors that enhance sensitivity and improve the selectivity of these films towards different gases [94-97]. The sensitivity of the WO_3_ sensing films deposited with interruptions by radio frequency (r.f.) sputtering onto silicon micro-machined substrates were higher than that obtained for the WO_3_ thin films deposited with basic technology due to the decrease of grain size in the WO_3_ films [95,96]. The sensors also show good selectivity to reducing gases. So, the results obtained showed that a decrease in grain size of the WO_3_-based sensing layer results in an increased sensitivity and selectivity to oxidizing gases [95,96].

WO_3_-based mixed oxides have also been investigated for their sensing characteristics. Modified materials include WO_3_-Ti [99-101], WO_3_-Pd, Pt, or Au [102-106], WO_3_-In_2_O_3_ [107], and WO_3_-Bi_2_O_3_ [108] which were used to fabricate selective and sensitive NOx gas sensors. For instance, sensors prepared based on semiconducting thin films of Ti, W, and Mo mixed oxides showed that the thin films had good sensing performances and the sensors were able to detect concentrations below the limit for environmental monitoring (CO, NO_2_) and breath analyzers (ethanol) [101]. Also, the sensitivity and selectivity of the deposited W–Ti–O mixed oxides thin films prepared using different Ti/W targets sputtered using an r.f. magnetron sputtering plant depend on the number and thickness of the Ti/W multilayers [99,100].

It was shown that the sensitivity, the minimum level of NO_x_ gas detection and the selectivity can be significantly improved by adding thin layers of noble metals such as palladium (Pd), platinum (Pt), and gold (Au) on the surface of the WO_3_ thin films operating at low sensor temperatures [102-106]. For example, pure and Au-doped WO_3_ powders prepared by a colloidal chemical method showed response values for NOx that depend on the operating temperature and the sensor's decomposition. The maximum gas response of the 1.5 wt.% Au-doped WO_3_ sensor was obtained at 200 °C while the 0.25, 0.5 and 1.0 wt.% Au-doped WO_3_ sensors gave the maximum gas response at 150 °C. Finally, physical vapor deposited Au-gates showed response to NO_2_ with positive flat-band-voltage shifts [107]. Response times were shorter than recovery times and were inversely related to gas concentration. At low NO_2_ concentrations, signal magnitude was limited by response time, whereas, at higher concentrations, the signal tended to saturate and the responses rapidly approached a steady state [107].

Platinum electrodes covered with Pt containing zeolite Y (PtY) and WO_3_ as the two electrode materials were examined [108]. Catalytic activity measurements and temperature programmed desorption showed that WO_3_ was almost inactive toward NO_x_ equilibration and no chemisorbed NO_x_ species was released from the WO_3_ surface. However, PtY had much higher activity towards NO_x_ equilibration. Due to this difference, compact solid-state potentiometric sensors were fabricated using PtY/Pt as the reference and WO_3_ as the sensing electrode. The use of a PtY filter made it possible to measure total NO_x_ in the sub-ppm level and the interferences from CO, propane, NH_3_, H_2_O and CO_2_ were minimized [108].

The role of Bi_2_O_3_ and indium additions to WO_3_ in the improvement of NO-sensitive properties of WO_3_ thick films, as well as the structure and gas-sensitive electrical properties of mixed WO_3_-Bi_2_O_3_ thick films were also examined [109,110]. It was found that the gas-sensitive properties of the WO_3_-Bi_2_O_3_ mixed thick films strongly depend on the Bi_2_O_3_ content. As the Bi_2_O_3_ content increases, the NO sensitivity of the WO_3_‐Bi_2_O_3_ thick films gradually deteriorates and eventually disappears. But, the WO_3_-Bi_2_O_3_ mixed thick films with Bi_2_O_3_ contents between 3–5 wt.% displayed a fairly good ability to detect NO in air in the range of 5–1,000 ppm at 350 °C [109]. Finally, indium-doped WO_3_ sensors were found to be more sensitive to NO_2_ when tested at 200 °C and more sensitive to CO when tested at 300 °C. The sensors showed the highest responsiveness to NO_2_ when the indium content was set at 3.0 wt. % [110]. Other studies have revealed that the gas sensors based on indium oxide nanowires and In_2_O_3_ thin films grown by the metal organic CVD technique showed good selectivity to NO_2_ with little interference from other gases [111-114].

**Figure 2.**
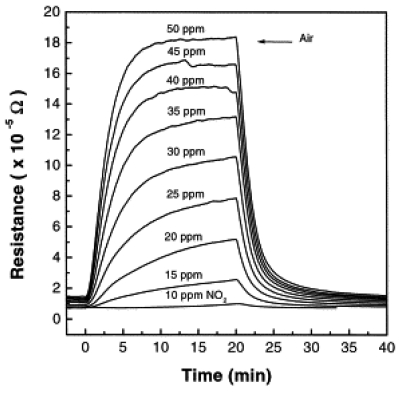
Electrical response of sprayed SnO_2_ thin films vs. NO_2_ concentration at 350 °C working temperature. (Reprinted from reference [139] with permission from Elsevier).

Investigators have also studied other SMO films such as SnO_2_ [103,115-126], ZnO [127-134], Te-oxide [135,136], Mo [137], gold [107], Pt [108], copper [138], and indium oxides [111,112]. Tin oxide thin films deposited onto different substrates such as Pyrex glass, Corning 7059 glass, and fused quartz showed a resistance change in the presence of 500 ppm of NO_2_ toxic gas at a working temperature of 350 °C and a sensitivity threshold of about 5 ppm at the same temperature [139]. An example of the electrical response of sprayed tin oxide thin films toward various concentrations of NO_2_ gas measured at 350 °C is presented in Figure 2. As shown in Figure 2, the device detects low concentration <10 ppm of NO_2_ with a clear gradual increase in the resistance as the concentration of the target gas increases [139].

In addition, the structural properties of polycrystalline Indium Tin Oxide (ITO) thin films were optimized in order to improve the stability of these nitrogen oxide detectors in the presence of high gas concentrations (1,000–2,000 ppm in air). It was found that ITO thin films exhibit high sensitivity toward NO_2_ and NO. Furthermore, they also exhibited good selectivity of these gases with respect to CO and CH_4_. It was also found that four zones for oxygen ion adsorption and desorption were able to be distinguished by a plot of conductivity activation energy vs. temperature which also established that nitrogen oxide desorption occurs at the same temperature (about 570 K) where O_2_^−^ desorption is supposed to take place [115].

ZnO sputtered thin films which were integrated with micro-arrays and deposited on Si [127] and Al [128] substrates were studied. The electrical response of the films to changes in concentration of NO_2_ along with other gases like H_2_, Liquified Petroleum Gas (LPG), H_2_S, CO were examined. ZnO films showed strong responses to even low concentrations of NO_2_ (1 ppm) and higher sensitivity at lower temperatures [127]. The gas sensing results on ZnO-Al films showed that the response increased with an increase in Al concentration up to 5 wt.% Al. It also showed that the response increased gradually with increasing NO_2_ concentration, and reached saturation at 100 ppm of NO_2_. At an operating temperature of 100 °C, the response towards lower NO_2_ concentrations is low irrespective of the Al concentration. While at 200 °C, the gas response was higher than that of 100 °C and reached saturation at around 150 ppm of NO_2_. At an operating temperature of 300 °C, the sensor was able to detect more than 150 ppm of NO_2_ [128].

Finally, TeO_2_ thin films were prepared by a reactive r.f. sputtering method and the NO_2_ gas sensing characteristics of these films were investigated [135,136]. The sensors were subjected to various concentrations of NO_2_ gas in the range of 1–120 ppm. The results showed the best sensitivity to NO_2_ at room temperature and the response decreased with an increase in working temperature. The response was found to be highest for films with a thickness of 300 nm, compared to those of 100 nm thickness. The response time was found to decrease with increasing gas concentration and it was about 6 min for 1 ppm to about 1.2 min for 120 ppm NO_2_ concentration. The recovery times, however, were longer than eight min for each gas concentration [135].

### 4.2. Sulfur dioxide detection

Sulfur dioxide is one of the typical air pollutants that must to be detected and then reduced in the environment by suitable methods. Many studies on the development of SO_2_ sensors have appeared, including liquid and solid electrolytes [140-146], as well as polymeric sensing films [147-153]. In contrast, only a few reports have been written on SMO sensing films for selective SO_2_ detection. Sensors based on SnO_2_ [154], SnO_2_ doped Pd [155], WO_3_ doped with various metals [156-158] and Vanadium oxide modified with TiO_2_ [158] were deposited and their sensing properties were measured and modified to reach a selective and sensitive detection level of SO_2_ gas. For example, Berger *et al.* [154] have reported the interaction mechanisms on the gas/sensor interface during the initial detection of sulfur dioxide were analyzed using results from the physico-chemical characterization of the SO_2_/SnO_2_ interaction. Surface acidity and the effects of SnO_2_ hydration were studied in order to show the effects of SO_2_ treatment. The results showed an increase in the density of the Lewis acidic sites after treating the samples with SO_2_. This increase was found to be dependent on temperature, with the highest value being obtained for a treatment temperature of 500 °C. This increase in density is assumed to be the reason for the sensor's increased sensitivity at high temperatures. It was also found that the irreversible formation of sulfate on the sensor surface is the cause of the irreversibility of the device's response after SO_2_ is first detected [154].

SnO_2_-based gas sensors containing 0.05, 0.1, 1, and 3 mol% Pd, as a catalytic additive, were fabricated using thick film technology and their response to CO gas was tested within a temperature range of 300 °C to 600 °C with either NO or SO_2_ being introduced as an interfering gas. The testing results showed that when SO_2_ was introduced, the response of the sensors toward CO increased up to a temperature of 450 °C after which it started to decrease when the temperature was raised to 500 °C, and further to 600 °C [155].

The potential of different WO_3_ based semiconductor metal oxides as SO_2_ sensors have been investigated [156-158]. Several attempts were made to improve the SO_2_ sensing properties of WO_3_ and SnO_2_ by the addition of a small amount of noble metals. Adding 1.0 wt.% of the metal to the WO_3_ powder was carried out by a conventional solution based method by employing HAuCl_4_·4H_2_O, AgNO_3_, Cu(NO_3_)_2_·3H_2_O, H_2_PtCl_6_·6H_2_O, PdCl_2_ and RhCl_3_·3H_2_O Each sensor material was mixed with a small amount of water and the resulting paste was applied to the surface of an alumina tube which had a pair of Pt wires serving as electrodes. It was then preheated to 950 °C for 10 hours in air prior to sensitivity measurements [156]. After the synthesis of the sensors, the sensitivity of the sensors from 200–800 ppm SO_2_ was measured in a flow apparatus in the temperature range of 100–800 °C. According to the experimental results, all the semiconductor metal oxides exhibited complex temperature- and time-dependant response curves for SnO_2_. However, among the oxides tested, WO_3_ exhibited the highest SO_2_ sensitivity at 400 °C, accompanied by a resistance increase, but its resistance to SO_2_ decreased at temperatures higher than 500 °C. Among the metals added to improve the SO_2_ sensitivity of WO_3_, the addition of 1.0 wt.% Ag was most effective for improving the sensitivity at 450 °C but also resulted in a decrease in sensor resistance upon exposure to SO_2_. When it came to cross selectivity, it was found that the resistance of WO_3_ increased upon exposure to both NO and NO_2_, and the NO_2_ sensitivity was superior to NO as well as SO_2_. In the case of 1.0 wt.% Ag/WO_3_, the results were similar but the interference from NO and NO_2_ was found to be more significant [156].

Active layers of pure and Pt doped WO_3_ were deposited using r.f. magnetron sputtering on micro-hotplate substrates and then their sensing properties to sulfur compounds (SO_2_ and H_2_S) were also investigated [157]. An integrated sensor containing an array of four microsensor elements was fabricated using microelectronic fabrication technology. The results showed that the sensors have high and reversible responses to the presence of H_2_S and SO_2_ diluted in CO_2_, in the absence of oxygen. Pure WO_3_ sensors were very sensitive to H_2_S, but not so for SO_2_. However the doped sensors showed the opposite behavior [157].

Recently, Liang *et al.* have modified a compact tubular sensor based on NASICON (sodium super ionic conductor) and a V_2_O_5_-doped TiO_2_ sensing electrode for the detection of SO_2_ [146]. The NASICON material was prepared from ZrO(NO_3_)_2_, NaNO_3_, (NH_4_)_2_HPO_4_ and Si(C_2_H_5_O)_4_ by a sol‐gel process. Nanometer-sized titanium dioxide was also prepared by a sol-gel method with Ti(OC_4_H_9_)_4_ as a precursor, C_2_H_5_OH as a solvent, and CH_3_COOH as a chelating reagent. NASICON was used as the basic material in the sensor and V_2_O_5_-doped TiO_2_ for the sensing electrode. The proportions of V_2_O_5_ to TiO_2_ were 0, 2, 5, 10 and 20 wt%. The sensors were exposed to sample gases containing different concentrations of SO_2_, NO, NO_2_, CH_4_, CO, NH_3_ and CO_2_ and their responses were measured. The results showed that the best sensing properties toward SO_2_ were shown by the sensor which had a thick film of NASICON and 5 wt% V_2_O_5_-doped TiO_2_ electrode sintered at 600 °C. The detection response time for 1–50 ppm SO_2_ was about 25–10 seconds while the recovery time was about 30–40 seconds. The sensor also showed excellent selectivity to SO_2_ against disturbing gases, and the operating temperature of the sensor was 300 °C [146].

### 4.3. H_2_S detection

SMO based sensors to detect H_2_S gas have received more attention than SO_2_ gases due to its toxic effects on human health. The threshold limit for H_2_S is 10 ppm. With concentrations above 250 ppm, H_2_S has a major effect on the human body, causing death. Since H_2_S occurs naturally in crude petroleum, natural gas, volcanic gases, as well as hot springs; and is generated by several industrial activities like bacterial decomposition of organic waste, food processing, cooking ovens, kraft paper mills, and petroleum refineries, the *in situ* monitoring of H_2_S is very important, especially in the industrial sector.

In recent years, studies on H_2_S detection and monitoring using SMO sensors have increased. The following SMO based sensors were successfully modified to selectively detect H_2_S: WO_3_ and WO_3_-based materials [159-165], SnO_2_ [166-171], ZnO [172,173], copper oxide [170,174,175], platinum and palladium oxides [176,177], indium oxides [177,178], silver based materials [169,179,180], titanium oxide [181] and cadmium oxide sensors [182].

WO_3_ based SMO sensors have received great attention for H_2_S detection. For example, WO_3_ films made by a r.f. deposition method employed in gas sensing showed that as-deposited films were sub-stoichiometric with various O/W ratios. The interaction with H_2_S was studied at 475 K, where the sensitivity of the film to the H_2_S gas is highest. The gas sensor's change in conductivity is most likely caused by the formation of a steady-state concentration of surface oxygen vacancies when the sensor is exposed to a given partial pressure of H_2_S in air [160]. Moreover, the H_2_S response properties of the WO_3_ thin film sensors were studied both in dry and wet synthetic air with different levels of humidity [161]. It has been noted that sputtered WO_3_ thin-film sensors give a large variation between the H_2_S response properties of sensors in the same sensor array where some sensors were found to be sensitive to H_2_S in the ppb range without gold doping, but with a slight increase in the conductance of the sensors in humid environments which interfere to some extent with the H_2_S sensing [161].

**Figure 3.**
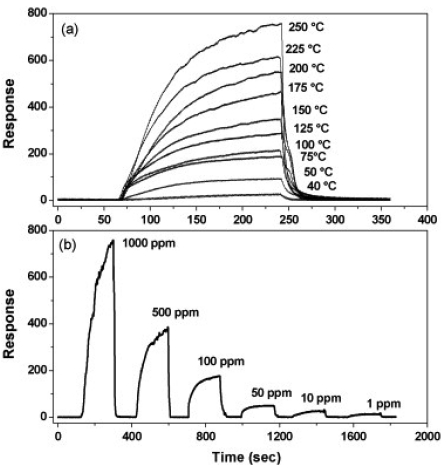
(a) Gas sensing characteristics of tungsten oxide nanoparticles to 1,000 ppm H_2_S, and (b) variations in response with concentration of H_2_S at 250 °C. (Reprinted from reference [165] with permission from Elsevier).

Unlike WO_3_ thin films, tungsten oxide nanostructures exhibit better sensing characteristics to H_2_S in the concentration range of 1–1,000 ppm over the temperature range of 40–250 °C. The best results were obtained with the WO_2.72_ nanowires at 250 °C where the response was not affected significantly up to 60% relative humidity (RH) [163,165]. A typical gas sensing profile of the above device toward the H_2_S detection at various temperatures and concentrations is shown in Figure 3. As shown in Figure 3, the highest response was observed at 250 °C with a possible detection limit under similar conditions that could reach the ppb range (<1 ppm) [165].

Recent studies showed that the amount of the dopant influences the sensitivity and the optimum operating temperature [159,164]. Among various dopants of gold, platinum, or palladium, it was found that the spillover effect of Pt dopant is larger than the gold dopant. In specific, under 1 ppm H_2_S and at an operating temperature of 220 °C, the individual sensitivities of the Pt and the Au-Pt doped WO_3_ gas sensors are 23 and 5.5, respectively. The results show that the Pt doped WO_3_ gas sensor exhibits acceptable response and recovery times, as well as a high sensitivity toward H_2_S [159,164].

Sols of crystalline SnO_2_ with various crystallite grain sizes ranging between 6 and 16 nm were prepared by subjecting stannic acid gel to hydrothermal treatments under various conditions. Thin film sensor devices with different film thicknesses between 200 and 900 nm were fabricated to investigate sensing properties toward H_2_S gas. It was found that the sensor response to H_2_S was significantly enhanced with decreasing film thickness and with increasing grain size up to 16 nm. The response was surprisingly large, exceeding 10^4^ at 150 °C, for the device deposited with a 200 nm hickness [166,167].

An Ag doped nanocrystalline SnO_2_ gas sensing material presents better sensitivity compared to pure SnO_2_, due to the distribution of Ag_2_O particles in grain boundaries of nanocrystalline SnO_2_ and the formation of p–n heterojunctions [168]. The H_2_S measurement results indicate that the developed of the H_2_S sensor's working temperature is about 70 °C, which is much less than commercially available sensors and recently developed SMO sensors [168]. Moreover, Cu-SnO_2_ composites show strong sensitivity toward H_2_S detection which reaches <10 ppm of H_2_S at a temperature of 100 °C [170,171]. Other sensors containing copper, iron, cadmium, and indium oxides were found to be selective toward H_2_S detection in ppm concentration levels [170,171,174-178,182]. Finally, both ZnO and tellerium oxide films were found to be highly sensitive to H_2_S gases at very low concentration levels [172,183]. For example, tellurium thin films prepared by thermal evaporation on alumina substrates at a temperature of 373 K were found to be sensitive towards 0.1 ppm of H_2_S at room temperature where hydrogen sulphide reduced the amount of adsorbed oxygen on the Te film surface leading to an increase in resistance [183]. Similarly, ZnO sensors fabricated from ZnO nanorods were found to be a suitable candidate for practical materials detecting low concentrations of H_2_S and C_2_H_5_OH where the sensors responded to 0.05 ppm H_2_S at room temperature [172].

### 4.4. NH_3_ and amine sensors

Detecting trace levels of ammonia is important since it is used extensively in many areas like food processing, fertilizers, chemical technology, medical diagnosis, and environmental protection. Some of the well known materials for ammonia sensors are WO_3_ [8,184], copper based materials [8,185], ZnO [186], SnO_2_ [187], iron oxide [188], Cr_2_O_3_ [189]. WO_3_ thin films were prepared via a sol-gel technique using WCl_6_ as a precursor and then tested for its sensing properties toward trimethylamine (TMA) gas at a low operating temperature of 70 °C. WO_3_ films were deposited between interdigital gold electrodes on the outer wall of a ceramic tube. The gas sensitivities to TMA, C_2_H_5_OH gas, gasoline, CH_4_, CO, and water vapor were measured. The sensitivity of the sensor was carried out in a range of temperatures and different TMA concentrations. For 100 and 500 ppm of TMA, the optimum operating temperature was found to be 70 °C. Even for 700 and 1,000 ppm concentrations of TMA, the sensitivity is highest at 70 °C [184].

Pure ZnO and RuO_2_-doped ZnO were prepared by a screen printing technique on an alumina substrate in a desired pattern and their gas sensing performances were studied. The thick film samples were made by dipping pure ZnO thick films into an aqueous solution (0.01 M) of ruthenium chloride for different time intervals: 5, 15, 30, 45 and 60 minutes [186]. The responses to 1,000 ppm NH_3_ of pure ZnO sensors fired at 500–700 °C were measured at operating temperatures between 100–350 °C. The response value increased with increasing operating temperature, and the sensor fired at 650 °C was the most sensitive. Variations in gas response to 1,000 ppm NH_3_ of ZnO films doped with different amounts of RuO_2_ and different operating temperatures were also measured [186]. In addition, ZnO thin films activated by chromic acid dipped for different time intervals and then fired at 500 °C for 24 hours in ambient air where CrO_3_ is not thermally stable above 197 °C and thus oxygen was lost, forming Cr_2_O_3_ which is a stable compound [189].

Cr_2_O_3_-activated sensors showed a good response to NH_3_ even at room temperature and were highly selective towards NH_3_ gas (300 ppm) even in the presence of other toxic gases of higher concentrations. The sensor also showed very rapid response and recovery times to NH_3_ gas [189]. In contrast, Cr_2_O_3_ thick films modified by 0.59 mass % Fe_2_O_3_ proved to be the most sensitive to not only NH_3_ gas but also LPG, C_2_H_5_OH and Cl_2_ gases [188]. The operating temperatures for NH_3_, C_2_H_5_OH, LPG, and Cl_2_ were found to be 250 °C, 300 °C, 400 °C, and 450 °C, respectively. It showed good selectivity to a particular gas at a particular temperature against other reducing gases. The sensor also showed very rapid response and recovery rates to reducing gases [188].

Gas sensitive sol-gel SiO_2_-SnO_x_-AgO_y_ films were fabricated where silver nitrate (AgNO_3_), 0.01 l.%, was added to tetraethoxysilane [(C_2_H_5_O)_4_Si] solutions mixed with stannic chloride (SnCl_4_·H_2_O), in a 5:1 ratio, in order to prepare an alcohol precursor. The 150-nm thick films were deposited by spin-coating on a silicon substrate. These obtained films were dried at 120 °C for 2 hours and then annealed at higher temperatures (from 350–600 °C) in air. The gas-sensitive properties of the films were tested to NH_3_ inputs which varied in the concentration range of 10–250 ppm in air. The films were shown to consist of Ag_2_O_3_, Ag_4_SiO_4_, Ag_2_SiO_3_, SnO, Sn_3_O_4_ and SnO_2_. It was confirmed that the response and recovery times depend on the Sn/Ag ratio. Further, an AFM study showed that the only films which were porous had a minimum Sn to Ag ratio of 0.5 and were annealed at 600 °C for eight hours, thus showing the best sensing characteristics. The films also showed good sensitivity to ammonia gas even at low temperatures (>50 °C) [190].

Finally, a different preparation technique for copper (I) bromide and their effects on its properties were investigated [185]. The two different techniques of preparation used were (1) magnetron sputtering (sensor A) and (2) electrochemical (sensor B1) or chemical (sensor B2) oxidation of copper in the presence of bromide ions. The detection of ammonia on CuBr sensors can be described as a two-step mechanism, involving the formation of a chemisorption layer during the ammonia treatment and dipolar effects due to physisorbed ammonia molecules during ammonia detection. All these results confirm that CuBr based sensors are of great interest for ammonia detection [185].

### 4.5. Hydrogen sensors

Hydrogen is a promising potential alternative fuel for automobiles and can be converted into electricity in fuel cells. It also is already used in medicine and space exploration as well as in the production of industrial chemicals and food products. Hydrogen sensors are needed because an explosive mixture can form if hydrogen leaks into air from storage tanks or valves. A nanostructured SnO_2_ thin film was fabricated by a spin coating together with a subsequent calcination process. Silver (Ag) and platinum (Pt) have been added as doping material in SnO_2_ to achieve better sensitivity and selectivity for H_2_ detection. The results of the tests showed that nanocrystalline SnO_2_ sensing films produced a fast response time of about two seconds and a quick recovery time of about 10 seconds with good sensitivity to hydrogen at 100 °C [191]. Porous SnO_2_ particles made using a Sol-gel method had higher sensitivity to H_2_ gas because of their high surface area [192]. A linear relationship between sensitivity and H_2_ concentration was observed on all sensors at an H_2_ concentration lower than 1,500 ppm. The results imply that there are potential applications for these high surface area SnO_2_ porous materials as highly sensitive sensors for the measurement of reducing gases at very low concentrations [192]. Moreover, a single wall carbon nanotube (SWCNT) reinforced nanocrystalline tin dioxide gas sensor was developed to achieve better gas sensing performance, in terms of sensitivity, response and recovery times, as well as a reduction in power consumption (low working temperature). Both the pure nano SnO_2_ sensor and the SWCNT/SnO_2_ sensor were tested in detecting various hydrogen concentrations [193]. The results showed that the SWCNT/SnO_2_ sensor's sensitivity for hydrogen detection was three times greater when compared to that of the pure SnO_2_ sensor over a hydrogen concentration range from 300 ppm to 1,500 ppm tested at a temperature of 250 °C [193].

Tungsten oxides supporting palladium or platinum catalysts were used as hydrogen-sensitive media. Their colors changed from pale green to blue when hydrogen reduces them to tungsten bronze [194]. Two different coatings of the WO_3_ were developed. In the first method, palladium-supported tungsten oxide powder was dispersed by dissolving tungsten oxide into a PdCl_2_ solution followed by annealing at 300 °C for 3 hours in air. The second sensor was developed using a Sol-Gel protocol in which, tungsten oxide sols are formed from the sodium tungstate aqueous solutions of various concentrations containing hydrogen tetrachloropalladate (II) acid (or chloroplatinic acid). The solution is acidified when it passes through a proton exchange resin [194]. The response time was greatly improved when the thin hydrogen-sensitive film was prepared by the sol–gel process where the sensor can measure the distribution along the fiber line, unlike the traditional hydrogen sensors that measure at a certain spatial point [194].

### 4.6. Ozone sensors

Ozone is one of the naturally occurring gases available in the atmosphere. However, a high level of ozone gas in the atmosphere is harmful to humans' respiratory system, causing inflammation and congestion of the respiratory tract [195]. This harmful level can result from the interaction between sunlight and various chemicals emitted into the environment by industrial means. Therefore, several materials based on WO_3_ [196-203] and SnO_2_ [204,205] have been fabricated to detect the ozone level in the atmosphere.

Novel sensors based on tungsten trioxide (WO_3_) semiconductors have been found to hold much promise as a cheaper alternative for ozone monitoring. For example, WO_3_ thin films deposited by reactive magnetron r.f. sputtering into silicon substrates have been investigated for ozone detection [196,197,200,202]. A clear enhancement of the sensor response to ozone was noticed when the grain size of the WO_3_ film decreases [202]. Recent studies have reported that the sensitivity of WO_3_ sensors strongly depends on working temperature, where at 573 K the sensor responses are the greatest [206]. The electrical properties of WO_3_ sputtered films depend upon the oxygen concentration during the deposition and during the resistivity versus temperature measurements. The activation energies are 0.19, 0.28 and 0.42 eV in the range of 300–723 K which indicates that the conduction mechanisms depend on oxygen concentration [196]. Further study on similar materials indicates that the adsorption efficiency in a mixture of air/ozone is strongly dependent on temperature as well [197]. Thus, the variation of the sensor's sensitivity with temperature is directly linked to the temperature dependence of the adsorption efficiency and the film morphology which strongly depends on the oxygen concentration during the deposition process [200].

WO_3_ based mixed oxide materials have also been investigated for ozone monitoring [199,201,203]. For example, the performances of three sensing layers — bare WO_3_, palladium, and gold activated surface WO_3_ — towards ethanol (C_2_H_6_O) and ozone (O_3_) were compared. Au has been found to be a good sensing activator for WO_3_ thin films. The sensitivities of Au/WO_3_ sensors to ethanol and ozone are in the 2/1 ratio; therefore, at 300 °C they can provide a stable, sensitive element for ethanol gas [199]. On the contrary, Pd/WO_3_ sensors are practically insensitive in this temperature range to the tested gases and could be used as selective elements against ozone [199]. Moreover, a small quantity of cobalt nanograins deposited on the surface of WO_3_ sensors produces a significant change in its conductance from n to p-type [203]. An increase in conductance of the WO_3_ sensors under ozone is thus observed.

Modified Co/W sensors have been tested under ozone before and after an annealing process under dry air at a temperature of 673 K for 1.5 hours [203]. The obtained response shape and mechanisms of ozone detection by Co/WO_3_ sensors suggest complex phenomena which depend on the strength of the metal substrate interaction and consequently could be induced by the formation of oxide species on the metal nanoparticles. To understand the changes that occur upon ozone exposure, a dynamic model based on the Wolkenstein adsorption theory has been developed [201]. The model suggested that the ozone detection mechanism of WO_3_-based gas sensors in dry air is essentially due to the adsorption of species O_2_, O_2_^−^, O and O^−^ at the surface of the grains. Both the simulation results and the experimental ones show good correlations [201].

A computerized Modular Ozone Sensor System (MOSS) based on various metal oxides (In_2_O_3_, SnO_2_) has been presented for evaluating the sensitivity and reliability of different sensor/transducer combinations. A material's sensitivity to ozone and its cross-sensitivity to other gases in ambient condition and to humidity were evaluated. It has been discovered that indium based materials had the largest sensor sensitivity as well as the smallest cross-sensitivities for ozone detection [204]. SnO_2_ films with a thickness of 30–200 nm deposited by spray pyrolysis shows a response to ozone that is quantitative and rapid and sufficient for use in ozone control and monitoring applications [205]. Sensor performance showed a large change in resistance upon exposure to ozone with maximum values for relative signals observed at an operating temperature ranges between 200–350 °C, (R_ozone_ /R_air_), in the range of 10^2^–10^4^ for ozone concentrations of ∼1 ppm in air at 35%–45% relative humidity (RH) [205].

### 4.7. Volatile organic compound sensors

Volatile organic compounds (VOCs) are very dangerous for both the environment and human beings. For humans, these compounds can cause many acute or chronic problems like eye irritation, throat and lung problems, as well as cancer. Therefore, during the past decade, several studies have been reported on modifying thin and thick film SMO sensors for atmospheric gaseous pollutants like VOCs. Several sensors have been fabricated during the last decade to selectively detect various VOC components like ethanol, acetone, hydrocarbon, and LPG. Some of these SMO sensors contain single metal or mixed metal oxides like SnO_2_ and SnO_2_-based materials [207-220], WO_3_ and WO_3_-based materials [221-224], titanium based oxides [225-227], zinc based oxides [214,224,228], iron based oxides [229,230], cobalt based oxides [231], cerium oxide sensor [232], and copper based materials [233].

When comparing the sensitivity of the SnO_2_ films, the ethanol gas sensitivity can be increased tremendously with an addition of a basic metal oxide such as La_2_O_3_ to SnO_2_. Ethanol gas undergoes dehydrogenation and dehydration over the SnO_2_-based elements loaded with a basic oxide (e.g., La_2_O_3_) and an acidic oxide (e.g., WO_3_), respectively [208]. As a result, SnO_2_ coated with a La_2_O_3_ layer using a 0.5 M La (NO_3_)_3_ aqueous solution showed an increase in response to acetone (∼3.6 times) and ethanol (∼5.5 times) with no variations in the responses toward propanol, CO, and H_2_ gases [211]. Moreover, tin oxide films doped with 2.0 wt.% CeO_2_ were found to dramatically improve sensitivity and selectivity to C_2_H_5_OH, in the presence of CO, LPG and CH_4_. The results show that ethanol selectivity is enhanced by factors of about 5.2, 5.3, and 48.2 with respect to CO, methane, and LPG, respectively. The enhancement in ethanol selectivity strongly depends on the temperature where the maximum selectivity is observed at 300 °C. At higher temperatures, its selectivity to ethanol sharply declines and the sensor becomes more selective to CO in the presence of ethanol and LPG [220].

Recently, the effect of CdO doping on the gas-sensing properties of SnO_2_-based sensors has been reported. Doping with CdO causes a remarkable improvement in sensitivities of SnO_2_ to C_2_H_5_OH and H_2_ with best sensitivity observed at 300 °C for the 10 mol% Cd-doped SnO_2_ film. The detection limit of this deposit is up to several ppm C_2_H_5_OH in air, making it applicable as a breath alcohol analyzer [210].

It has been noted that the mode of deposition of thin tin based oxide films highly influenced their physical, electrical, and chemical properties [207,209,212-214,234]. For example, SnO_2_–In_2_O_3_ nanocomposites fabricated with a coprecipitation method achieved superb response to ethanol by tuning the content of indium [209]. In addition, sensors prepared by mixing the SnO_2_ paste with a Pt paste before firing, showed sensitivity to ethanol that was five times higher than one of the sensors prepared by a r.f. magnetron sputtering method. The 3% Pt-doped samples have an extremely high sensitivity to ethanol vapors and their responses are linear in the ppb range with a detection limit below 1 ppb at an operation temperature of 300 °C [212].

Finally, several tin oxide based films have been modified to detect other VOCs like vapors of LPG, acetylene, and aldehyde with high sensitivity and selectivity. A SnO_2_–NiO composite material provides a stable and sensitive film for detecting low concentrations of HCHO [215]. Despite the fact that the response and recovery time of the film sensor decreases rapidly with an increase in the HCHO concentration; at relatively low concentrations, the micro-gas sensor can detect 0.06 ppm HCHO and shows high selectivity in the presence of interference gases, such as acetone, alcohol, -pinene and toluene, which makes it promising for the detection of indoor HCHO [215]. Qi *et al.* have reported that 6 wt% Sm_2_O_3_-doped SnO_2_ displays a superior response for C_2_H_2_ that is 16.8 times larger than that of pure SnO_2_ at an operating temperature of 180 °C. This sensor also shows high sensitivity under various humid conditions which make it a good candidate for fabricating C_2_H_2_ sensors [216]. SnO_2_ based sensors have been modified to detect LPG [217,219]. For example, it has been reported that SnO_2_ sputtered with Pt, Ag, Ni, and Pb using a r.f. technique show good detection toward LPGs. Among all of these devices, the SnO_2_–Pt-dotted island structure exhibits enhanced the response for LPG at a relatively low operating temperature of 260 °C. The presence of Pt islands on the SnO_2_ film results in enhanced sensing characteristics with a fast response speed (about 100 s) and a fast recovery time (about 120 s) [217]. Moreover, the gas sensitivity of the SnO_2_ gas sensor toward LPG was improved by Al doping, which is further improved by Ni doping due to a significant reduction in the grain size of the composite material [219].

TiO_2_ and W/TiO_2_ thin films with increasing W content deposited via a spin-coating method presented high ethanol sensing performances [222]. Doping with W resulted in an increased response with respect to pure TiO_2_ where, spin-coated W/TiO_2_ thin films showed a very high ethanol response compared with those already presented for TiO_2_ [235]. In addition, Nb-Pt co-doped TiO_2_ and the hybrid single wall carbon nanotubes (SWCNTs)/Nb-Pt co-doped TiO_2_ thin films prepared by the sol-gel spin-coating process have been tested for ethanol detection [225]. The results revealed that the responses to ethanol of the Nb–Pt co-doped TiO_2_ sensors with SWCNTs inclusion increase by factors of 2–5 depending on the operating temperature and the ethanol concentration, compared to that of the sensor without SWCNT inclusion with a maximum sensitivity and stability at 335 °C [225].

WO_3_ thick films prepared by a screen-printing method exhibited excellent acetone vapor sensing properties with a maximum sensitivity reached at 300°C along with fast response and recovery times. Further, the screen printed WO_3_ thick films can be reliably used to monitor the concentration of acetone vapor over the concentration range of 25–75 ppm [223]. The response and recovery characteristics of the WO_3_ thick films are reproducible and quick. Thus, this study demonstrates the possibility of utilizing WO_3_ thick films as a sensor element for the detection of acetone vapor [223]. Interestingly, Cr_2−_*_x_*Ti*_x_*O_3_ (*x* = 0–0.5, CTO) powders prepared by a combustion technique [227] showed a linear increase in the sensor response to acetone as a function of concentration. The quick response and recovery of these materials indicate their potential as excellent candidates for acetone monitoring. The exponential decay of the sensor relative response comes to a constant value after 80 hours of exposure to 1 ppm of acetone which indicates that the sensor could operate for several hundred hours with outstanding performance and continuous usage [227].

A nanosized ZnO powder was synthesized by using a chemical precipitation method, and loaded with different dopants (Ru, Mg, Pd,Y, La,V, and Na) through impregnation. The prepared ZnO powder shows excellent gas responses to alcohol and acetaldehyde with no response to ethene. In addition, among all the dopants, Ru is the optimal dopant that can increase the sensor's response to C_2_H_5_OH [228]. Various metal oxides were modified by doping with lanthanum have been reported as selective VOC sensors. For example, the perovskite-type nano-crystalline thin-film of LaFeO_3_ obtained by using a sol-gel coating technique proved to be a good ethanol sensor that could be used as a detector for a wide range of C_2_H_5_OH concentrations between 100–1,000 ppm with excellent stability [229].

Nominal La_1−_*_x_*Mg*_x_*FeO_3_ (*x* = 0 – 0.7) nano-powders were prepared using a sol–gel method. It has been noted that for samples La_1−_*_x_*Mg*_x_*FeO_3_, the Mg composition *x* affects the structure, resistance in air, and gas sensing properties to methane gas. The resistance of La_1−_*_x_*Mg*_x_*FeO_3_ is smaller than that of LaFeO_3_ with *x* ≤ 0.1. The La_0.9_Mg_0.1_FeO_3_-based sensor shows a high response to methane gas, low operating temperature, and excellent stability in air [230]. However, in the presence of interfering gases like methanol and CO, the sensor lacks selectivity. Recently, LaCoO_3_ perovskite has been modified as an active filter for eliminating the sensor's sensitivity to CO and ethanol [231]. Both CO and ethanol are completely removed by the filter at temperatures as low as 190 °C. At 250 °C, the sensor's sensitivity to ethanol dramatically decreased from 158 to 0.44 and that to CO declined from 2.2 to 0.9, when an active filter is used. Therefore, only methane reaches the Pt/SnO_2_ sensor at temperatures higher than 190 °C, for which the sensor shows high sensitivity to methane. As a result, the LaCoO_3_ perovskite filter eliminates the sensor sensitivity to CO and ethanol, making the sensor highly selective to methane in the presence of CO and ethanol in air [231].

### 4.8. Chemical warfare agents

The identification and quantification of chemical warfare agents (CWAs) in the battlefield and public areas is extremely important to eliminate the threat of these chemicals to humans. Various spectroscopic techniques have been employed to detect CWAs such as NMR [236-246], Mass Spectrometry [247-251], Gas Chromatography-Mass spectroscopy (GC-MS) [252], Fourier Transform Infrared (FTIR) spectrometry [253-257], Raman spectroscopy [258,259], Atomic emission and Flame photometry [260], Ion mobility spectrometry [261-266], and Fourier Transform microwaves [267]. However, the high cost and complexity of the sampling and detection procedures make it extremely important to develop devices that will provide real time, sensitive, and selective CWA detection with low cost. Several studies have shown that surface acoustic wave polymers provide highly selective sensors for CWA detection [268-278] but they still lack sensitivity. For the last three decades, semiconducting metal oxides (SMOs) such as SnO_2_, WO_3_, and ZnO have been extensively studied for sensing hydrocarbon and other chemical agents. The major problem with SMO sensor technology is that it lacks selectivity. Therefore, various semiconducting metal oxides such as In_2_O_3_, TiO_2_, WO_3_, CuO have been developed for enhancing selectivity to a particular analyte.

Tin oxide based gas sensors have been used to detect the toxic gases and chemical agent simulants [9,15,279-284]. For example, tin oxide nanowires prepared by a vapor-liquid-solid deposition method was found to be very sensitive to acetonitrile and dimethyl methyl phosphonate, DMMP, the most commonly used simulant molecule for sarin. The modified materials were found to be useful to detect concentrations of both simulant CWAs at concentrations lower than the respective CWAs Immediately Dangerous to Life or Health (IDLH) values [279]. Lee *et al.* [281] have previously reported that the particle and the pore size, as well as the doping of the sensing materials play significant roles in the sensitivity of the SMO particles [281]. In specific, during the detection of acetonitrile and dichloromethane on tin oxide thick film sensors, the sensor prepared from a small SnO_2_ precipitated particle (15 nm) was more sensitive than that prepared from the commercial SnO_2_ (40 nm) with a significant enhancement in the sensor's sensitivity upon doping with NiO or Nb_2_O_5_. The sensitivity of the SnO_2_ sensor linearly increased in acetonitrile between 0.02 and 0.2 ppm at 350 °C whereas, for other chemical agent simulants, the sensitivity increased linearly between 0.1 and 0.8 ppm [281]. The recovery of the sensors seemed to be possible for acetonitrile whereas, in the cases of DMMP and dichloromethane, the complete recovery of the sensor was not possible because of poisoning. However, addition of Sb_2_O_3_ or MoO_3_ dopants enhanced the recovery of the sensors after the exposure of DMMP or dichloromethane [281]. The same research group has recently reported that three components; namely, Mo, Sb, and Ni (Mo_5_Sb_1_·Ni(I)) promoted the SnO_2_ based sensors for the detection of DMMP [280]. In specific, the Mo_5_Sb_1_·Ni(I) sensor showed not only an excellent sensor response in the detection of a very low concentration of DMMP (ppb level), but also a complete recovery. Also, the Mo_5_Sb_1_·Ni_2_(I) sensor developed in this study showed a high sensor response of about 70% in the detection of 0.5 ppm DMMP at 350 °C [280]. Finally, it has been reported that adding basic oxides like CaO and MgO to SnO_2_ or ZnO-based elements exhibit a reasonable sensitivity to DMMP down to 44 ppb. The role of basic oxide additives aids the dissociative adsorption of DMMP on the oxide surface, thus facilitating the oxidation reaction of the test gas [282].

Nano-sized high surface area WO_3_ powders have shown high sensitivity toward CWA detection, especially the DMMP simulant molecule [285-291]. It has been noted that DMMP adsorbs on the high surface area TiO_2_ and WO_3_ powders through hydrogen bonding of the P=O functional group to the hydroxyl groups of the metal oxide surface. At higher reaction temperatures, these hydrogen bonded organophosphorous compounds dissociate and form covalently attached species. Above 200 °C, the methoxy groups desorb from the surface while the methyl groups remain stable. Above 300 °C, a stable phosphate surface complex is formed and causes poisoning effects observed during DMMP gas exposure of chemiresistive sensors operating in this temperature range [291]. Moreover, a WO_3_ based chemiresistive sensor has been designed and tested for chlorine detection with high sensitivity (as low as 0.05 ppm) and a short response time (< 1 minute) [290]. The modified sensor is small, portable, inexpensive, and may have applications as an element in a chemical warfare sensing array [290].

Besides developing new semiconducting metal oxides, compositions of binary or polynary SMOs have been optimized for improved selectivity of target analytes. For example, Quan reported that trinary composition of SnO_2_-In_2_O_3_-TiO_2_ with some trace dopants (Pd, Al, Si, *etc.*) enhanced the selectivity toward combustion-type CH_4_ gas at high concentration (≥500 ppm) [292]. More recently, SMO hybrid materials with non-conducting inorganic oxides as well as SMO surface modification by noble/transition metals or metal oxides have been widely reported for enhancing sensitivity and selectivity toward the target chemical agents. For example, surface-modified tin oxide by ruthenium and palladium oxides has improved sensitivity to hydrogen at high concentration (1,000 ppm) [293,294], tin oxide modified by ruthenium or surface-ruthenated tin oxide has improved selectivity to hydrocarbon at high concentration (1,000 ppm) [295], tin oxide modified by CuO has high selectivity to H_2_S at 200 °C [296], surface-modified indium oxide by Rb_2_CO_3_ gives a surprisingly selective detection of CO [297], CaO or MgO surface-modified tin oxides exhibit promising sensing properties to dimethyl methylphosphonate (DMMP) [282]. Therefore, the modified SMO sensors can selectively detect target analytes at high concentration, but at low concentration (<10 ppm), SMO sensors may lack selectivity for practical CWA applications.

**Figure 4.**
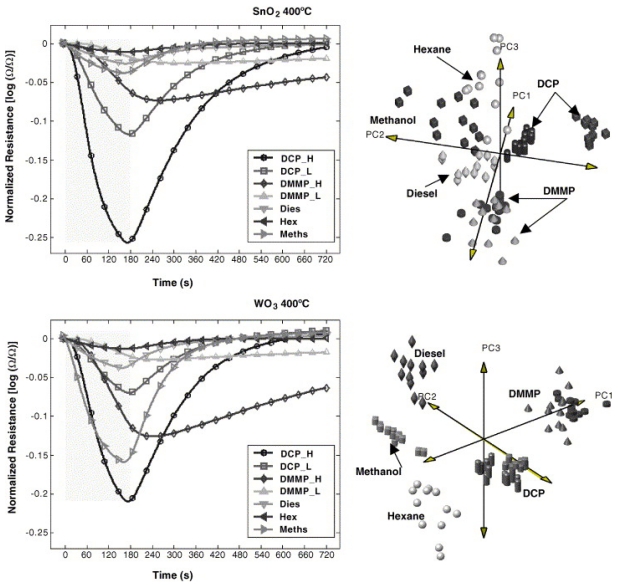
SMO sensors' normalized responses to the gases of interest and corresponding LDA. (Reprinted from reference [13] with permission from Elsevier).

Recently, several studies have focused on providing sensitive and selective CWA detection. Methods including a combination of filtration, concentration, and array based detection have been reported [287,298-301]. Materials such as inorganic membranes, zeolites, and other adsorbents are used to selectively preconcentrate and prefilter interferent molecules from the gas stream [299-304]. An array-based approach increases the information content of the response signal because each element of the array produces a different response characteristic to the gas matrix. In this case, a bank of sensors is used in which each sensor element produces a different response to the various components of the gas stream [13,14]. Variables such as metal oxide composition and morphology, impregnation with metal catalysts and operational temperature are a few approaches that are under investigation to achieve distinguishable sensor array elements [305-310]. For example, thick films of various SMO components prepared via drop-coating techniques followed by annealing using an internal heater in the sensor platform have been studied on various real CWAs, CWA simulants, as well as interfering gaseous molecules [13]. The study showed that nano-sized materials based on WO_3_, SnO_2_, and In_2_O_3_ can detect low levels of CWAs in the ppb range within a short time. For example, Figure 4 depicts an example of the SnO_2_ and WO_3_ thick film sensors for CWA detection at 400 °C. In addition, using significant differences occur during either the physical or the chemical adsorption processes on the SMO films, one can discriminate between various gases on the same sensor platform by applying suitable pattern recognition techniques like linear discriminant analysis (LDA) or principal component analysis (PCA) [13,14]. As shown in Figure 4 (right graphs) the WO_3_ based device, has the potential to discriminate between various gases based on variations in molecular structure that affect the target-surface reaction protocols and thus lead to differences in the response shape [13].

**Figure 5.**
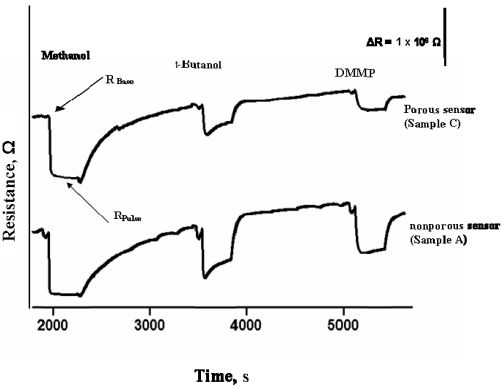
Sensor response to a three pulse sequence of methanol, t-butanol and DMMP for samples A and C based sensors. (Reprinted from reference [285] with permission from Elsevier).

A simpler and direct protocol has been recently reported by Kanan *et al.* to selectively discriminate DMMP from a gas stream [285,286]. The method established a unique selective and size sensitive sensor array for CWA detection using dual a sensor configuration that is coated with porous and nonporous WO_3_ nano-material. By comparing the sensor response on a porous WO_3_ powder (samples B and C) to the response on a nonporous WO_3_ powder sensor (sample A), detection selectivity between methanol and DMMP was obtained because the access of a gas molecule in the interior pore structure of WO_3_ is size dependent; thus leading to a size dependant magnitude change in the resistance of the SMO sensor. Several responses have been recorded for methanol and DMMP along with a series of alcohols of different shapes and sizes in order to demonstrate the size selective detection [285]. Figure 5 shows a typical sensor response to three consecutive gas pulses of methanol, *t*-butanol, and DMMP on porous and nonporous based sensors [285]. As shown in Figure 5, the response of the porous sensor to DMMP is weaker than the response of the nonporous based sensor for the same target gas (DMMP). In contrast, the responses of both sensors toward methanol look similar because methanol has a small size so it can access the pore of the porous material [285].

The change in conductivity (ΔC) obtained on each porous WO_3_ sensor is then ratioed to the corresponding ΔC obtained on the nonporous WO_3_ sensor (ΔC_porous_/ΔC_nonporous_) which provided a clear distinction between methanol and DMMP, which is larger in size compared to methanol [285].

## 5. Concluding Remarks

As discussed in this review, in our opinion, there are several important and potentially existing sensor arrays for selective and sensitive detection for each of the studied toxic pollutants. Therefore, such systems can have a major impact on human health and safety for domestic use as well as, various industrial and homeland security. Below is a summary of distinguished SMO based sensors for specific pollutant detection:

- Carbon nanotubes modified with SMO materials like WO_3_ composites can detect ppb concentration levels of nitrogen oxide (NO or NO_2_) gases at room temperature [90,91].
- Tin oxide thin films as well as tin oxide and tungsten oxide doped with noble metals like platinum and gold provide sensitive SO_2_ detection but these devices operate at temperatures above 350 °C [139,156,157].
- A tin oxide thin film with 200 nm thickness was found to be a highly sensitive sensor toward H_2_S at 150 °C [166,167]
- A Cr_2_O_3_-based sensor provided sensitive and selective detection for gaseous ammonia at room temperature [189].
- SnO_2_ doped silver or platinum provided a unique sensor for H_2_ detection [192].
- WO_3_ films represent good devices for ozone detection where the films thickness, grain size, and operating temperatures have to bse adjusted to reach an optimal response [202,206].
- SnO_2_ doped with basic oxides like La_2_O_3_ and CdO provide a unique response toward alcohol in air making it applicable as alcohol breath detectors [210].
- Several SMO based materials have been used to detect sarin-like stimulants (DMMP) in ppb concentration levels but limitations like recovery and selectivity still need to be resolved. Several methods were applied to enhance selectivity including filteration, size selective detection, as well as pattern recognition techniques [13,280,285,293, 300,304].
